# Association of diet quality with hand grip strength weakness and asymmetry in a multi-ethnic Asian cohort

**DOI:** 10.1017/S0007114523002647

**Published:** 2024-04-14

**Authors:** Jiannan Huang, Aarathi Shanmugam, Xiangyuan Huang, Rob M. van Dam, Saima Hilal

**Affiliations:** 1 Saw Swee Hock School of Public Health, National University of Singapore and National University Health System, 117549 Singapore; 2 Departments of Exercise and Nutrition Sciences and Epidemiology, Milken Institute School of Public Health, The George Washington University, Washington, DC, USA; 3 Department of Pharmacology, Yong Loo Lin School of Medicine, National University of Singapore, Singapore

**Keywords:** Sarcopenia, Grip strength, Diet quality, Asia, Multi-ethnic population

## Abstract

Hand grip strength (HGS) is an important diagnostic tool for sarcopenia and a reliable predictor for age-related chronic diseases and mortality. Interventions in nutrition have been shown as a low-cost strategy to maintain muscular strength and mass. However, there are limited data on the effect of diet on HGS in Southeast Asian populations. This study aims to investigate the association of diet quality with HGS weakness and asymmetry in a multi-ethnic population in Singapore. This cross-sectional study used data from the Singapore Multi-Ethnic Cohort (*n* = 1547). Dietary data were collected using a validated semi-quantitative FFQ and summarised as the Dietary Quality Index – International (DQI-I). HGS was calculated as the maximum value of six measurements from both hands. HGS weakness and asymmetry were defined using well-recognised criteria. Multivariable linear regression and logistic regression were utilised for continuous and binary outcomes, respectively, adjusting for age, sex, ethnicity, physical activity and smoking status. It was found that the highest quartile of DQI-I was significantly associated with higher HGS (*β* = 1·11; 95 % CI 0·41, 1·82; *P*_for trend_ < 0·001) and lower odds of HGS asymmetry (OR = 0·71; 95 % CI 0·53, 0·94; *P*_for trend_ = 0·035) and both HGS weakness and asymmetry (OR = 0·50; 95 % CI 0·32, 0·76; *P*_for trend_ = 0·004). Among the different components of DQI-I, only dietary adequacy was significantly associated with higher HGS (*P*_for trend_ < 0·001) and lower odds for both HGS weakness and asymmetry (*P*_for trend_ = 0·006). Our findings support that DQI-I, an indicator of overall diet quality, can be used to provide dietary guidelines for prevention and management of muscle wasting, sarcopenia and frailty.

## Introduction

According to the WHO, the proportion of adults above 60 years of age is expected to increase exponentially worldwide^(1)^. Frailty and sarcopenia, which are associated with morbidity and mortality in older adults^(2)^, have considerable overlap in characteristics, including decrease in grip strength, gait speed, muscle mass and functional mobility^(3)^. These characteristics are associated with adverse health outcomes such as an increased risk of falls, institutionalisation and mortality^(4,5)^.

The European Working Group on Sarcopenia in Older People (EWGSOP) and the Asian Working Group for Sarcopenia (AWGS) have recommended cut-off values for hand grip strength (HGS), physical performance and muscle mass measurements as screening tests of sarcopenia, but there is no consensus on the age range for sarcopenia screening^(6,7)^. Since WHO’s Framework for healthy ageing recommends that early-stage interventions are crucial to delaying frailty^(8)^, it is worth examining populations younger than the current recommended guidelines (> 60 years) because loss of muscle mass and strength is pronounced even around the age of 50 years^(9)^.

HGS is a convenient and reliable measure of strength capacity and is recommended by both EWGSOP and AWGS for the assessment of muscle strength^(6,7)^. HGS weakness, defined as HGS lower than specific cut-off values, has been verified to be associated with chronic diseases, disabilities and mortality^(10)^. Moreover, HGS asymmetry, determined by asymmetric grip strength between hands, has also been shown to be a potential predictor of future accumulating morbidities^(11)^, falls^(12)^ and mortality^(13)^.

Poor nutrition, especially inadequate intake of energy and protein, plays a key role in the pathogenesis of muscle atrophy, leading to a decrease in muscle strength and mass^(6)^. Interventions focusing on nutrition assessment may be feasible and cost-effective for maintaining muscle strength and mass at a population level.

The measure of dietary quality, which reflects overall dietary patterns, may be better in capturing the health effects of diet than single nutrients or foods^(14)^. The Dietary Quality Index – International (DQI-I) includes both nutrient and food groups, considering the variety, adequacy, moderation and balance of individuals’ diets^(14)^. Higher DQI-I scores have been associated with better health outcomes such as a lower prevalence of sarcopenia, a decreased prevalence of obesity and a reduced risk of metabolic syndrome^(15,16)^.

While most research on the association between nutrition and HGS has been conducted in Western countries, only a few studies have focused on Asian populations^(15,17,18)^. However, these results were all based on single East Asian populations and are therefore non-representative of the ethnic diversity in Southeast Asian countries. Furthermore, these studies were restricted to older adults. Therefore, large-scale observational studies are needed to assess the effect of nutrition on HGS in a multi-ethnic Southeast Asian population with a wider adult age range.

In this study, we aim to investigate the association between diet, using DQI-I as an indicator, and HGS (including HGS weakness and asymmetry) in a multi-ethnic population in Singapore. We hypothesise that individuals with high dietary quality have increased HGS as well as lower odds of HGS weakness and asymmetry in this multi-ethnic population.

## Methods

### Study design and participants

This study used cross-sectional data from the follow-up of the Singapore Multi-Ethnic Cohort (MEC)^(19)^. The cohort was followed up between 2011 and 2016, comprising 6101 participants (aged 21–75 years and consisting of Chinese, Malay, and Indian ethnicities). During this revisit, FFQ and HGS measurements were conducted.

**Fig. 1.**
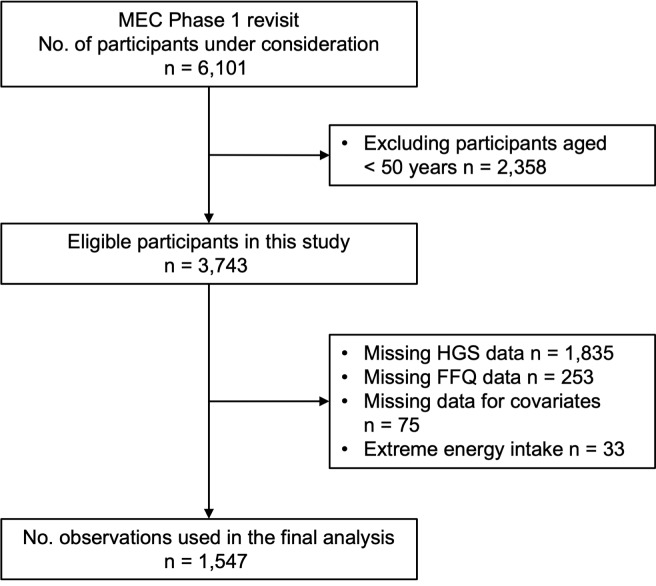
The flow chart of eligible and excluded Singapore Multi-Ethnic Cohort (MEC) participants in this study. HGS, hand grip strength.

Of the 6101 participants, those aged 50 years or over were enrolled in this study (*n* 3743). Further exclusion criteria included individuals with missing data for HGS (*n* 1835), FFQ (*n* 253) and covariates (*n* 75). Individuals with extreme energy intakes ( < 500 kcal or > 8000 kcal; *n* 33) were also excluded, leaving 1547 participants for final analysis (Fig. 1).

### Calculation of Dietary Quality Index – International scores

Dietary intake was assessed using a semi-quantitative 169-item FFQ, developed from a 2-d 24-h dietary recall survey. This recall survey covered dietary habits on both a working day and a non-working day in the local population^(20,21)^. Each participant’s FFQ recorded the frequency of consuming one serving of each item over the past year, along with a description of the standard portion size for each item. The recorded frequency for each item was then converted into the number of servings per d and totalled within five food groups, including vegetables, fruits, grains, meat/poultry/fish/egg and dairy products/beans. To estimate daily energy intake and daily consumption of macronutrients and micronutrients for each participant, we utilised the Singapore Food Composition database (accessible at https://focos.hpb.gov.sg/eservices/ENCF/)^(19)^. This database provided information on energy content and the amounts of various macronutrients and micronutrients per serving of each food item.

Dietary data from the FFQ were converted into DQI-I, which has four components: variety, adequacy, moderation and balance (online Supplementary Table 1). Based on predefined scoring criteria, each component of DQI-I is awarded a score and DQI-I is calculated by adding the four scores together, generating a score ranging from 0 to 100. A higher score represents better diet quality^(14)^. In this study, Na and cholesterol calculations were omitted because Na intake remained difficult to quantify in Singapore, and dietary cholesterol was indicated as an inaccurate predictor of health outcomes by recent studies^(22,23)^. Therefore, the possible maximum DQI-I score in this study was 88. DQI-I was used as both a continuous and categorical variable (divided into quartiles, with the 1st quartile having the lowest diet quality). Moreover, DQI-I component scores were divided into tertiles with the 1st tertile having the lowest score. Of note, the numbers of participants across tertiles of DQI-I components were not equal because usually more than one-third of participants had the same score and were divided into the same tertile.

### Measurement of hand grip strength

HGS was measured using a hand dynamometer (TAKEI A5401), and three readings were recorded for each hand. HGS (kg) was calculated as the maximum value of six measurements from both hands. HGS (kg) was firstly treated as a continuous outcome variable. Thereafter, to examine the association of DQI-I with HGS weakness and asymmetry, three binary outcomes were assessed separately, including HGS weakness alone, HGS asymmetry alone, and both HGS weakness and asymmetry. The cut-off values for HGS weakness were defined as < 28 kg for males and < 18 kg for females according to the AWGS 2019 consensus^(6)^. Based on the ‘10 % rule’, HGS asymmetry was defined as the ratio of maximum HGS for non-dominant hand to that for dominant hand below 0·90 or over 1·10^(11)^. HGS weakness and asymmetry were treated as a composite outcome if the criteria for both HGS weakness and HGS asymmetry were met.

### Assessment of covariates

Smoking status was coded as a binary variable (never smoking or ever smoking). Diabetes was defined based on any of the following criteria: 1. formerly diagnosed by a physician; 2. taking anti-diabetic medication; 3. fasting blood glucose ≥ 7·0 mmol/l; 4. random blood glucose ≥ 11·1 mmol/l.

A physical activity questionnaire was used to record the type, frequency and duration of various activities performed by participants in different domains, including transportation, occupation, leisure time and household activities. The daily physical activity level (MET-h/d) was calculated using the 2011 Compendium of Physical Activity based on the responses provided in the questionnaire^(24)^. The physical activity questionnaire was validated for use in the local population and demonstrated good to modest agreement with accelerometer measurements^(19)^. However, given the potential for error in assessing low-intensity activities, only activities of moderate-to-vigorous activity (MVPA), defined as activities over 3·0 metabolic equivalents (MET), were considered in the analysis.

### Statistical analysis

Baseline characteristics of participants were expressed as mean (sd) or median (25^th^ and 75th percentile) for continuous variables based on data distribution and as count (percentage) for categorical variables. To compare differences in baseline characteristics across DQI-I quartiles, Pearson’s *χ* ^2^ test, Kruskal–Wallis test, and ANOVA were conducted for categorical, non-normally distributed, and normally distributed variables, respectively.

Multivariable linear regression was used to estimate the regression coefficients (*β*) and 95 % CI for the association between DQI-I and HGS (kg). The association between each DQI-I component and HGS (kg) was also examined using linear regression. Collinearity of covariates with DQI-I was evaluated by generalised variance inflation factor of each covariate using package Car in R. Multivariable logistic regression was used to estimate the OR and 95 % CI for the association between DQI-I and odds of HGS weakness, HGS asymmetry, or both. The dose–response relationship between the quantiles of DQI-I or DQI-I components and HGS was evaluated using *P*_for linear trend_.

Multivariable regression model building was performed using a forward stepwise approach, and the entry criterion for covariates was set as a *P*-value of < 0·1 in the simple regression model. Furthermore, covariates that could be on the causal pathway between DQI-I and HGS (e.g. total energy intake, BMI and diabetes) were excluded. Model 1 was an unadjusted model. Model 2 adjusted for age (years), sex, ethnicity, total MVPA (MET-h/d) and smoking status.

To determine whether age, sex, ethnicity and physical activity modified the association between DQI-I and HGS, multiplicative interaction terms of DQI-I with these factors were added in the fully adjusted model^(25,26)^.

To control for the oversampling strategy employed in the MEC study, which means the Malay and Indian participants were oversampled as compared with the general population, a weight of 1·637, 0·625 and 0·237 was assigned to Chinese, Malay and Indian participants, respectively, based on the ethnic distribution. Subsequently, weighted regression analyses were conducted which showed similar relationship of DQI-I with HGS (online Supplementary Table 2–4).

Statistical analysis was performed using R (version 4.2.1, R Foundation for Statistical Computing, Vienna, Austria). Statistical significance was defined as a *P*-value of < 0·05.

## Results

### Characteristics of study participants

Table 1 shows the baseline characteristics of study participants in each quartile of DQI-I scores. The mean (sd) age of participants included in our study was 59·2 (7·5) years, and 58·9 % of participants were female. The mean (sd) DQI-I score was 35·8 (12·3) out of 88, and the mean (sd) HGS was 25·1 (8·1) kg. Participants with higher DQI-I scores were more likely to be female, of Chinese and Indian ethnicities, and have higher physical activity, total energy intake, and higher HGS. BMI, smoking status and proportions of diabetes were not significantly different across DQI-I quartiles.

**Table 1.** Baseline characteristics of study participants by dietary quality (DQI-I) score*

Characteristics			DQI-I quartiles								*P*_for trend_
	Overall (*n* 1547)		Q1 (*n* 388)		Q2 (*n* 360)		Q3 (*n* 397)		Q4 (*n* 402)		
	*n*	%	*n*	%	*n*	%	*n*	%	*n*	%	
DQI-I score											
Mean	35·8		19·9		31·2		40·1		51·1		< 0·001
sd	12·3		4·8		2·5		2·6		4·9		
Age (years)											
Mean	59·2		58·6		59·2		59·3		59·6		0·27
sd	7·5		7·1		7·6		7·5		7·7		
Female	911	58·9	264	68·0	210	58·3	205	51·6	232	57·7	< 0·001
Ethnicity											0·005
Chinese	559	36·1	132	34·0	130	36·1	145	36·5	152	37·8	
Malay	437	28·3	139	35·8	97	26·9	92	23·2	109	27·1	
Indian	551	35·6	117	30·2	133	36·9	160	40·3	141	35·1	
Ever smoker	338	21·9	77	19·9	84	23·3	102	25·7	75	18·7	0·07
Physical activity (MET-h/d)											
Median	6·5		5·4		6·2		6·4		8·0		< 0·001
25th, 75th percentile	3·0, 12·8		2·5, 11·5		2·8, 12·8		3·0, 12·4		4·0, 14·4		

	Mean	sd	Mean	sd	Mean	sd	Mean	sd	Mean	sd	
BMI (kg/m^2^)	26·2	5·2	26·7	5·7	26·1	5·2	25·8	4·8	26·0	5·0	0·11
Diabetes (yes)											
*n*	326		79		90		81		76		0·20
%	21·1		20·4		25·0		20·4		18·9		
Total energy intake (kcal/d)	2082·1	760·8	1645·3	557·3	1879·7	580·5	2185·0	688·4	2583·2	823·8	< 0·001
Hand grip strength (kg)	25·1	8·1	23·5	7·6	24·7	8·0	26·3	8·3	25·7	8·2	< 0·001

DQI-I, Dietary Quality Index – International; MET, metabolic equivalents of task.

* Values are expressed as mean (sd) or median (25th, 75th percentile) for continuous variables based on data distribution and as n (%) for categorical variables. Pearson’s *χ* ^2^ test, Kruskal–Wallis test and ANOVA were conducted for categorical, nonnormal and normally distributed variables, respectively.

Due to a large proportion of participants with missing HGS data, we compared baseline characteristics between participants with and without HGS data (online Supplementary Table 5). The results suggest that these two groups are largely comparable.

### Associations of Dietary Quality Index – International with hand grip strength (kg)

As shown in Table 2, each sd increment in DQI-I was associated with an increase in mean HGS by 0·51 (95 % CI 0·26, 0·76) kg in the fully adjusted model. Higher quartile of DQI-I was significantly associated with higher HGS (*P*_for trend_ < 0·001), and highest quartile showed 1·11 (95 % CI 0·41, 1·82) kg higher HGS than the lowest quartile after adjustment.

**Table 2.** Association of dietary quality (DQI-I) with hand grip strength (kg) as a continuous variable*

Nutrition Index	Model 1			Model 2		
	*β*	95 % CI	*P*	*β*	95 % CI	*P*
DQI-I, per sd	1·00	0·60, 1·40	< 0·001	0·51	0·26, 0·76	< 0·001
DQI-I quartiles						
Q1	Ref			Ref		
Q2	1·20	0·05, 2·35	0·041	0·11	–0·61, 0·83	0·76
Q3	2·78	1·65, 3·91	< 0·001	0·92	0·21, 1·62	0·011
Q4	2·20	1·07, 3·32	< 0·001	1·11	0·41, 1·82	0·002
*P*_for trend_	< 0·001			< 0·001		

DQI-I, Dietary Quality Index – International; MVPA, moderate-to-vigorous physical activity; MET, metabolic equivalents of task.

* Model 1: unadjusted model. Model 2: adjusted for age (years), sex, ethnicity, total MVPA (MET-h/d) and smoking status.

### Associations of Dietary Quality Index – International with hand grip strength weakness and asymmetry

After adjustment for covariates, each sd increment in DQI-I was associated with 15 % lower odds of HGS weakness (OR = 0·85, 95 % CI 0·75, 0·95), 10 % lower odds of HGS asymmetry (OR = 0·90, 95 % CI 0·81, 0·99) and 23 % lower odds of both HGS weakness and asymmetry (OR = 0·77, 95 % CI 0·66, 0·90) (Table 3).

**Table 3.** OR for the association of dietary quality (DQI-I) with hand grip strength weakness and asymmetry*

Nutrition Index	HGS weakness						HGS asymmetry						HGS weakness and asymmetry					
	Model 1			Model 2			Model 1			Model 2			Model 1			Model 2		
	OR	95 % CI	*P*	OR	95 % CI	*P*	OR	95 % CI	*P*	OR	95 % CI	*P*	OR	95 % CI	*P*	OR	95 % CI	*P*
DQI-I, per sd	0·87	0·78, 0·97	0·016	0·85	0·75, 0·95	0·007	0·89	0·80, 0·98	0·022	0·90	0·81, 0·99	0·039	0·77	0·67, 0·90	< 0·001	0·77	0·66, 0·90	0·001
DQI-I quartiles																		
Q1	Ref			Ref			Ref			Ref			Ref			Ref		
Q2	0·88	0·64, 1·20	0·40	0·86	0·61, 1·20	0·37	0·57	0·42, 0·76	< 0·001	0·57	0·43, 0·77	< 0·001	0·60	0·40, 0·90	0·014	0·59	0·38, 0·90	0·015
Q3	0·76	0·56, 1·04	0·09	0·75	0·53, 1·04	0·09	0·60	0·45, 0·79	< 0·001	0·61	0·46, 0·81	< 0·001	0·62	0·42, 0·92	0·017	0·64	0·42, 0·97	0·036
Q4	0·73	0·54, 1·00	0·049	0·69	0·50, 0·97	0·033	0·69	0·52, 0·92	0·011	0·71	0·53, 0·94	0·017	0·49	0·32, 0·74	< 0·001	0·50	0·32, 0·76	0·001
*P*_for trend_	0·033			0·023			0·022			0·035			0·002			0·004		

DQI-I, Dietary Quality Index – International; MVPA, moderate-to-vigorous physical activity; MET, metabolic equivalents of task.

* Hand grip strength weakness was defined as maximum hand grip strength below sex-specific cut-offs (< 28 kg for males and < 18 kg for females). Hand grip strength asymmetry was defined as the ratio of maximum hand grip strength for non-dominant hand to that for dominant hand below 0·9 or over 1·1. HGS weakness and asymmetry was treated as a composite outcome if the criteria for both HGS weakness and HGS asymmetry were met. Model 1: unadjusted model. Model 2: adjusted for age (years), sex, ethnicity, total MVPA (MET-h/d) and smoking status.

Compared with the lowest quartile, the highest DQI-I quartile observed 31 % lower odds of HGS weakness (OR = 0·69; 95 % CI 0·50, 0·97; *P*_for trend_ = 0·023), 29 % lower odds of HGS asymmetry (OR = 0·71; 95 % CI 0·53, 0·94; *P*_for trend_ = 0·035), and 50 % lower odds for both HGS weakness and asymmetry (OR = 0·50; 95 % CI 0·32, 0·76; *P*_for trend_ = 0·004), in the fully adjusted model.

### Association of hand grip strength with individual Dietary Quality Index – International components

Table 4 displays the association of each DQI-I component included in this study with HGS (kg) and HGS weakness and asymmetry. Compared with the lowest tertile, a mean increase of 1·47 (95 % CI 0·66, 2·28) kg in HGS was observed in the highest tertile of adequacy and the dose–response relationship across the tertiles was significant (*P*_for trend_ < 0·001), after adjusting for demographics and lifestyle factors. Moreover, the highest tertile of adequacy showed 51 % lower odds for both HGS weakness and asymmetry (OR = 0·49; 95 % CI 0·29, 0·81; *P*_for trend_ = 0·006) compared with the lowest tertile. However, associations of HGS with other components of DQI-I (variety, moderation and balance) were not significant.

**Table 4.** Association of dietary quality score (DQI-I) components with hand grip strength*

DQI-I components	Tertile	*n*	HGS (kg)			HGS weakness and asymmetry		
			*β*	95 % CI	*P*	OR	95 % CI	*P*
Variety	T1	748	Ref			Ref		
	T2	523	–0·08	–0·74, 0·58	0·81	1·06	0·71, 1·59	0·77
	T3	276	–0·39	–1·26, 0·47	0·37	1·34	0·77, 2·29	0·29
*P*_for trend_					0·37			0·29
Adequacy	T1	580	Ref			Ref		
	T2	525	0·48	–0·18, 1·14	0·16	0·62	0·41, 0·92	0·020
	T3	442	1·47	0·66, 2·28	< 0·001	0·49	0·29, 0·81	0·006
*P*_for trend_					< 0·001			0·006
Balance	T1	557	Ref			Ref		
	T2	556	–0·27	–0·86, 0·32	0·37	0·91	0·63, 1·29	0·59
	T3	434	0·58	–0·16, 1·33	0·12	0·68	0·43, 1·06	0·09
*P*_for trend_					0·12			0·09
Moderation	T1	627	Ref			Ref		
	T2	421	–0·45	–1·11, 0·22	0·19	1·35	0·91, 1·99	0·13
	T3	499	–0·09	–0·79, 0·62	0·81	0·95	0·61, 1·46	0·80
*P*_for trend_					0·81			0·80

DQI-I, Dietary Quality Index – International; MVPA, moderate-to-vigorous physical activity; MET, metabolic equivalents of task.

* Model adjusted for age (years), sex, ethnicity, total MVPA (MET-h/d) and smoking status. Hand grip strength weakness was defined as maximum hand grip strength below sex-specific cut-offs (< 28 kg for males and < 18 kg for females). Hand grip strength asymmetry was defined as the ratio of maximum hand grip strength for non-dominant hand to that for dominant hand below 0·9 or over 1·1. HGS weakness and asymmetry was treated as a composite outcome if the criteria for both HGS weakness and HGS asymmetry were met.

### Assessment of effect modifiers in the association between Dietary Quality Index – International and hand grip strength

There was no significant interaction between DQI-I and HGS by age, sex, and physical activity. However, the association between DQI-I and HGS (kg) was significantly different across ethnicity (*P*_for interaction_ = 0·011).

We further stratified the data by ethnic groups (online Supplementary Table 6). We found that there was no significant relationship between DQI-I and HGS (kg) in the Malay, while a significant association was observed between DQI-I and HGS (kg) in Chinese and Indian ethnicities (for Chinese, *β* = 0·57, 95 % CI 0·16, 0·98; for Indian, *β* = 0·95, 95 % CI 0·47, 1·43). Moreover, there was a significant association of DQI-I with HGS weakness (OR = 0·73, 95 % CI 0·59, 0·89), HGS asymmetry (OR = 0·81, 95 % CI 0·67, 0·96), and both HGS weakness and asymmetry (OR = 0·66, 95 % CI 0·51, 0·85) in the Indian ethnicity.

## Discussion

In this multi-ethnic Southeast Asian population, higher adherence to a healthy dietary pattern measured by DQI-I was associated with higher HGS and lower odds of HGS weakness and asymmetry, independent of demographics and lifestyle factors. Sufficient consumption of vegetables, fruits, protein and other beneficial foods and nutrients might be the most critical factor of a healthy diet that could help improve HGS.

Nutrition has long been considered important for maintaining muscle strength. In a cross-sectional study of a US adult population, the highest quartile of Healthy Eating Index-2015 (HEI-2015) was associated with a 24 % lower risk of having low HGS compared with the lowest quartile of the study population^(27)^. Another US study used three indices to evaluate diet quality and found that those in the highest quintile of diet scores had 53 % lower odds of frailty and 25 % lower odds of pre-frailty compared with the lowest quintile^(28)^. A UK study in an elderly population indicated that poor diet quality was significantly related to lower HGS at baseline and follow-up^(29)^. Besides the aforementioned studies which were performed in Western countries, some other studies have explored the relationship between nutrition and muscle strength in single East Asian populations. A prospective cohort study in a Chinese elderly population in Hong Kong indicated a 50 % reduction in the risk of sarcopenia (only for men) in the highest quartile of DQI-I compared with the lowest^(15)^ and a 31 % reduction in the risk of frailty per 10-unit increase in DQI-I scores^(17)^. Another study in a Korean elderly population found that higher scores of dietary quality indices were associated with lower odds of low HGS^(18)^. Therefore, the direct association between diet quality and HGS (kg) and the inverse association between diet quality and odds of HGS weakness in our study are consistent with previous findings.

The previous studies that examined the relationship between diet and muscle strength usually focused on dietary patterns because this could help assess the overall influence of the diet and take into account potential interactions between dietary constituents^(30)^. This approach was illustrated by a systematic review of prospective cohort studies, which showed that high adherence to the Mediterranean and Nordic diet was associated with a lower risk of sarcopenia^(31)^. The Mediterranean and Nordic diet are both characterised by a high consumption of vegetables, fruits, wholegrain products, and plant oils, a low consumption of red meat, and low to moderate intake of alcohol^(27)^.

The association between the consumption of specific foods and nutrients with muscle strength has been investigated in observational and intervention studies^(32,33)^. Dietary protein intake, as an important nutrient to promote muscle protein synthesis, is crucial for the maintenance of muscle mass and strength^(34)^. In the prospective Framingham Offspring Cohort, increased protein intake was significantly associated with higher HGS, regardless of the protein source^(35)^. Another study using UK Biobank data also found a direct association between protein intake and HGS^(34)^. Other food groups such as vegetables and fruits are considered beneficial for maintaining muscle function, because they contain a wide range of antioxidants (e.g. vitamin C, vitamin E and polyphenols). Oxidative damage of muscle proteins may be a key pathological mechanism leading to muscle strength loss, especially in elderly populations^(36)^. A cross-sectional study in Korean older adults showed that a higher consumption of vegetables and fruits was inversely associated with the risk of sarcopenia^(37)^. Another study in the UK using increased consumption of vegetables and fruits as an intervention indicated that higher intake of vegetables and fruits could improve muscle strength in healthy older adults^(38)^. Interestingly, both studies took into account the total amount of daily intake of vegetables and fruits rather than the diversity of vegetables or fruits consumed.

Our study showed a similar pattern when we further investigated the impact of each DQI-I component on HGS. Among the four components comprising DQI-I (variety, adequacy, moderation and overall balance), only adequacy was found to be significantly associated with HGS as well as odds of HGS weakness and asymmetry. This suggests that sufficient consumption of vegetables, fruits, protein and other beneficial foods and nutrients is important to maintain muscle strength in this study population. Eight dietary variables are considered in the adequacy component of the DQI-I: vegetables, fruits, grains, fibre, protein, Fe, Ca and vitamin C^(14)^. Each dietary variable is given a score of 5 or 0 based on a cut-off value, which indicates sufficient intake for a healthy diet. As discussed above, previous studies support that higher intakes of vegetables, fruits, protein and vitamin C are beneficial for maintaining muscle strength. A higher intake of these nutrients will reflect as a higher score for the DQI-I adequacy component. In our study, dietary variety was not significantly associated with HGS, neither overall variety across food groups nor variety for protein source. These findings suggest that sufficient intake of beneficial foods and nutrients may matter more than variety for the maintenance of HGS. This finding is consistent with previous studies which showed the benefits of adequate consumption of protein, vegetables and fruits for muscle strength regardless of diversity within these food groups^(35,37,38)^.

Furthermore, our study was the first to examine the association between diet quality and HGS asymmetry and the results showed a probable quadric relationship with the second quartile of DQI-I having the lowest odds of HGS asymmetry. We cannot fully exclude the possibility that some covert confounders could have biased this relationship, leading to a non-linear association. This speculation requires further verification.

In this study, we did not find the association between DQI-I and HGS in the Malay. This might be attributed to genetic differences across ethnicities. Moreover, dietary patterns differ vastly across ethnic cultures^(39)^, and these differences may be difficult to capture and evaluate in diet questionnaires. No previous studies have compared the association between diet quality and HGS across ethnicities, and therefore our study sheds light on a possibility that ethnicity may influence the effect of diet on muscle strength, which requires investigation in large-scale and multi-ethnic observational studies in the future.

This study has some limitations worth mentioning. As a cross-sectional study, we can only confirm an association between diet quality and HGS rather than causality. Only HGS was collected in this cohort without measurements on physical performance or muscle mass, so only the relationship of diet quality with muscle strength rather than sarcopenia could be investigated. Dietary information was collected via self-reported questionnaires, which might lead to some misclassification. Furthermore, two categories (i.e. cholesterol and Na) were omitted when calculating moderation scores (6 points for each) in this study, and it might slightly alter the measures of association between overall DQI-I scores and HGS. Another limitation of this study is residual confounding, which is common in an observational study. However, we adjusted for a wide range of potential confounders in regression models, including demographics and lifestyle factors. Although we find an association between muscle strength and adequate intake of beneficial foods and nutrients, we cannot examine the individual components (e.g. protein) and provide a recommendation for their daily intake. Therefore, further studies focusing on the consumption of certain foods and nutrients may be of concern.

This study used data from a health screening cohort, including three major ethnic groups in Singapore with a broad age range, which provides a high degree of generalisability for the results and thus is one of the most important strengths of this study. This study is also a complement to current findings regarding the association between diet quality and HGS in Southeast Asian populations. Our dataset included many covariates related to diet quality and muscle strength, facilitating adjustment for confounders. Moreover, HGS is considered a convenient measure of assessing sarcopenia owing to its low cost, ease of use, and its association with leg strength^(40)^ and therefore can be implemented as part of a comprehensive screening tool in primary care settings. Likewise, DQI-I can be used widely to assess nutrition status using a short screener with good reproducibility and relative validity^(21)^. These features make it feasible and practical to use DQI-I as a dietary criterion to guide people how to follow a healthy diet and prevent sarcopenia.

### Conclusion

In a multi-ethnic Southeast Asian population, higher dietary quality was associated with better muscle strength measured by HGS and lower odds of HGS weakness and asymmetry. In particular, sufficient consumption of vegetables, fruits, protein, and other beneficial foods and nutrients was associated with higher HGS and also lower odds of HGS weakness and asymmetry. Our findings support that DQI-I, an indicator of overall diet quality, can be used to evaluate nutrition status of people and provide dietary guidelines for prevention and management of muscle wasting, sarcopenia, and frailty.

## Supporting information

Huang et al. supplementary material 1 Huang et al. supplementary material

Huang et al. supplementary material 2 Huang et al. supplementary material

Huang et al. supplementary material 3 Huang et al. supplementary material

Huang et al. supplementary material 4 Huang et al. supplementary material

Huang et al. supplementary material 5 Huang et al. supplementary material

Huang et al. supplementary material 6 Huang et al. supplementary material

## Supplementary material

For supplementary material accompanying this paper visit https://doi.org/10.1017/S0007114523002647
