# Supplementary material for: Association of diet quality with hand grip strength weakness and asymmetry in a multi-ethnic Asian cohort
Source: Br J Nutr. 2023 Nov 22;131(7):1236–43. doi: 10.1017/S0007114523002647 (PMC10918521; doi:10.1017/S0007114523002647)
Supplement: Huang et al. supplementary material 1 — Huang et al. supplementary material [file S0007114523002647sup001.docx]

Supplementary Table 1. Dietary Quality Index - International (DQI-I) components and scoring in this study

| Component | scoring criteria | score |
| --- | --- | --- |
| Variety |  | 0-20 points |
| Overall food group variety (meat/poultry/fish/egg; dairy/beans; grains; fruits; vegetables) | ≥1 serving from each food group/d = 15  Any 1 food group missing/d = 12  Any 2 food group missing/d = 9  Any 3 food group missing/d = 6  ≥4 food group missing/d = 3  None from any food groups = 0 | 0-15 points |
| Within-group variety for protein source (meat, poultry, fish, dairy, beans, eggs) | ≥3 different sources/d = 5  2 different sources/d = 3  From 1 source/d = 1  None = 0 | 0-5 points |
| Adequacy |  | 0-40 points |
| Vegetables group | ≥3–5 servings/d = 5, 0 servings/d = 0 | 0-5 points |
| Fruit group | ≥2–4 servings/d = 5, 0 servings/d = 0 | 0-5 points |
| Grain group | ≥6–11 servings/d = 5, 0 servings/d = 0 | 0-5 points |
| Fiber | ≥20–30 g/d = 5, 0 g/d = 0 | 0-5 points |
| Protein | ≥10% of energy/d = 5, 0% of energy/d = 0 | 0-5 points |
| Iron | ≥100% RDA (AI)/d = 5, 0% RDA (AI)/d = 0 | 0-5 points |
| Calcium | ≥100% AI/d = 5, 0% AI/d = 0 | 0-5 points |
| Vitamin C | ≥100% RDA (RNI)/d = 5, 0% RDA (RNI)/d = 0 | 0-5 points |
| Moderation* |  | 0-18 points |
| Total fat | ≤20% of total energy/d = 6  >20-30% of total energy/d = 3  >30% of total energy/d = 0 | 0-6 points |
| Saturated fat | ≤7% of total energy/d = 6  >7-10% of total energy/d = 3  >10% of total energy/d = 0 | 0-6 points |
| Empty calorie foods | ≤3% of total energy/d = 6  >3-10% of total energy/d = 3  >10% of total energy/d = 0 | 0-6 points |
| Overall balance |  | 0-10 points |
| Macronutrient ratio (carbohydrate:protein:fat) | 55 ~ 65:10 ~ 15:15 ~ 25 = 6  52 ~ 68:9 ~ 16:13 ~ 27 = 4  50 ~ 70:8 ~ 17:12 ~ 30 = 2  Otherwise = 0 | 0-6 points |
| Fatty acid ratio (PUFA:MUFA:SFA) | P/S = 1 ~ 1.5 and M/S = 1 ~ 1.5 = 4  Else if P/S = 0.8 ~ 1.7 and M/S = 0.8 ~ 1.7 = 2  Otherwise = 0 | 0-4 points |
| DQI-I scores in this study |  | 0-88 points |

Abbreviations: RDA, Recommended Dietary Allowance; AI, Adequate Intakes; RNI, Recommended Nutrient Intake; PUFA, polyunsaturated fatty acids; MUFA, monounsaturated fatty acids; SFA, saturated fatty acids; P/S, ratio of PUFA to SFA intake; M/S, ratio of MUFA to SFA intake.

*Sodium and cholesterol (originally included as indices for moderation) were omitted in this study as sodium intake remains difficult to quantify in Singapore, and recent research has indicated that dietary cholesterol is not an accurate predictor of health outcomes.
