# Supplementary material for: Association of diet quality with hand grip strength weakness and asymmetry in a multi-ethnic Asian cohort
Source: Br J Nutr. 2023 Nov 22;131(7):1236–43. doi: 10.1017/S0007114523002647 (PMC10918521; doi:10.1017/S0007114523002647)
Supplement: Huang et al. supplementary material 2 — Huang et al. supplementary material [file S0007114523002647sup002.docx]

Supplementary Table 2. Association of dietary quality (DQI-I) with hand grip strength (kg) as a continuous variable after weighting by ethnicity*

| **Nutrition Index** | **Model 1** | | **Model 2** | |
| --- | --- | --- | --- | --- |
|  | β (95% CI) | p value | β (95% CI) | p value |
| DQI-I, per SD | 1.05 (0.65, 1.44) | <0.001 | 0.47 (0.23, 0.71) | <0.001 |
| DQI-I quartiles |  |  |  |  |
| Q1 | Ref | | Ref | |
| Q2 | 1.68 (0.55, 2.82) | 0.004 | 0.05 (-0.64, 0.75) | 0.88 |
| Q3 | 2.87 (1.76, 3.98) | <0.001 | 0.72 (0.04, 1.40) | 0.037 |
| Q4 | 2.77 (1.67, 3.86) | <0.001 | 1.19 (0.51, 1.87) | <0.001 |
| p-trend | <0.001 | | <0.001 | |

Abbreviations: DQI-I, Dietary Quality Index - International; MVPA, Moderate-to-Vigorous Physical Activity; METs, Metabolic equivalents of task.

*Model 1: unadjusted model. Model 2: adjusted for age (years), sex, ethnicity, total MVPA (MET-hrs/d), and smoking status.
