# Supplementary material for: Association of diet quality with hand grip strength weakness and asymmetry in a multi-ethnic Asian cohort
Source: Br J Nutr. 2023 Nov 22;131(7):1236–43. doi: 10.1017/S0007114523002647 (PMC10918521; doi:10.1017/S0007114523002647)
Supplement: Huang et al. supplementary material 5 — Huang et al. supplementary material [file S0007114523002647sup005.docx]

| Supplementary Table 5. Baseline characteristics between participants with and without HGS data* | | |
| --- | --- | --- |
| **Characteristics** | **With HGS data**  **(n = 1,908)** | **Without HGS data**  **(n = 1,835)** |
| Age, years, mean (SD) | 59.2 (7.4) | 60.3 (8.1) |
| Female, n (%) | 1,115 (58.4) | 1,053 (57.4) |
| Ethnicity, n (%) |  |  |
| Chinese | 703 (36.8) | 1,117 (60.9) |
| Malay | 528 (27.7) | 320 (17.4) |
| Indian | 672 (35.2) | 394 (21.5) |
| Ever smoker, n (%) | 422 (22.1) | 371 (20.2) |
| BMI, kg/m^2^, mean (SD) | 26.1 (5.1) | 24.8 (4.1) |
| Abbreviations: HGS, Hand grip strength; BMI, Body mass index.  *Values expressed as mean (SD) or median (25th, 75th percentile) for continuous variables based on data distribution and as n (%) for categorical variables. | | |
