# Supplementary material for: Association of diet quality with hand grip strength weakness and asymmetry in a multi-ethnic Asian cohort
Source: Br J Nutr. 2023 Nov 22;131(7):1236–43. doi: 10.1017/S0007114523002647 (PMC10918521; doi:10.1017/S0007114523002647)
Supplement: Huang et al. supplementary material 6 — Huang et al. supplementary material [file S0007114523002647sup006.docx]

Supplementary Table 6. Association of dietary quality (DQI-I) with hand grip strength stratified by ethnicity*

| **Ethnicity** | **N** | **HGS (kg)** | | **HGS weakness** | | **HGS asymmetry** | | **HGS weakness and asymmetry** | |
| --- | --- | --- | --- | --- | --- | --- | --- | --- | --- |
|  |  | **β (95% CI)** | **p value** | **OR (95% CI)** | **p value** | **OR (95% CI)** | **p value** | **OR (95% CI)** | **p value** |
| Chinese | 559 | 0.57 (0.16, 0.98) | 0.006 | 0.84 (0.67, 1.04) | 0.11 | 1.05 (0.88, 1.25) | 0.58 | 0.90 (0.67, 1.22) | 0.51 |
| Malay | 437 | 0.06 (-0.36, 0.48) | 0.78 | 1.01 (0.81, 1.26) | 0.94 | 0.83 (0.69, 1.00) | 0.048 | 0.83 (0.63, 1.09) | 0.19 |
| Indian | 551 | 0.95 (0.47, 1.43) | <0.001 | 0.73 (0.59, 0.89) | 0.003 | 0.81 (0.67, 0.96) | 0.018 | 0.66 (0.51, 0.85) | 0.001 |

Abbreviations: DQI-I, Dietary Quality Index - International; MVPA, Moderate-to-Vigorous Physical Activity; METs, Metabolic equivalents of task.

*Model adjusted for age (years), sex, total MVPA (METs-h/d), and smoking status. DQI-I z-scores were used in this analysis. Hand grip strength weakness was defined as maximum hand grip strength below gender-specific cut-offs (< 28 kg for males and < 18 kg for females). Hand grip strength asymmetry was defined as the ratio of maximum hand grip strength for nondominant hand to that for dominant hand below 0.9 or over 1.1. HGS weakness and asymmetry was treated as a composite outcome if the criteria for both HGS weakness and HGS asymmetry were met.
